# The Queensland experience of participation in a national drug use evaluation project, Community-acquired pneumonia – towards improving outcomes nationally (CAPTION)

**DOI:** 10.1186/1471-2466-9-38

**Published:** 2009-08-03

**Authors:** Lisa K Pulver, Susan E Tett, Judith Coombes

**Affiliations:** 1 School of Pharmacy, Steele Building, University of Queensland, Queensland, Australia; 2 Faculty of Health Sciences, University of Queensland, Edith Cavell Building, Royal Brisbane and Women's Hospital, Herston, Queensland, Australia; 3 Pharmacy Department, Princess Alexandra Hospital, Ipswich Rd, Woolloongabba, Queensland, Australia

## Abstract

**Background:**

Multicentre drug use evaluations are described in the literature infrequently and usually publish only the results. The purpose of this paper is to describe the experience of Queensland hospitals participating in the Community-Acquired Pneumonia Towards Improving Outcomes Nationally (CAPTION) project, specifically evaluating the implementation of this project, detailing benefits and drawbacks of involvement in a national drug use evaluation program.

**Methods:**

Emergency departments from nine hospitals in Queensland, Australia, participated in CAPTION, a national quality improvement project, conducted in 37 Australian hospitals. CAPTION was aimed at optimising prescribing in the management of Community-Acquired Pneumonia according to the recommendations of the Australian Therapeutic Guidelines: Antibiotic 12th edition. The project involved data collection, and evaluation, feedback of results and a suite of targeted educational interventions including audit and feedback, group presentations and academic detailing.

A baseline audit and two drug use evaluation cycles were conducted during the 2-year project. The implementation of the project was evaluated using feedback forms after each phase of the project (audit or intervention). At completion a group meeting with the hospital coordinators identified positive and negative elements of the project.

**Results:**

Evaluation by hospitals of their participation in CAPTION demonstrated both benefits and drawbacks. The benefits were grouped into the impact on the hospital dynamic such as; improved interdisciplinary working relationships (e.g. between pharmacist and doctor), recognition of the educational/academic role of the pharmacist, creation of ED Pharmacist positions and enhanced involvement with the National Prescribing Service, and personal benefits. Personal benefits included academic detailing training for participants, improved communication skills and opportunities to present at conferences. The principal drawback of participation was the extra burden on already busy staff members.

**Conclusion:**

A national multicentre drug use evaluation project such as CAPTION allows hospitals which would otherwise not undertake such projects the opportunity to participate. The Queensland arm of CAPTION demonstrated benefits to both the individual participants and their hospitals, highlighting the additional value of participating in a multicentre project of this type.

## Background

Few true multicentre Drug Use Evaluations (DUEs) are described in the literature [1]. The CAPTION (Community-Acquired Pneumonia (CAP) Towards Improving Outcomes Nationally) project was designed as a national quality improvement project, with concurrent drug use evaluations of the management of CAP in the Emergency Department (ED) being conducted in 37 hospitals across Australia [2]. The project was supported and funded by the National Prescribing Service (NPS) Ltd, an independent, non-profit member-based organisation providing accurate, balanced, evidence-based information and services to health professionals and the community on Quality Use of Medicines (QUM)[3]. The NPS, established in 1997, is funded by the Australian Government Department of Health and Ageing.

Some larger hospitals in Australia provide a drug use evaluation program, run mainly by pharmacists or clinical pharmacologists (where available); however these programs essentially run independently at an individual hospital level. Until recent years, the NPS focused on community prescribing issues, but it is recognised that prescribing may be initiated in hospital and continued in the community. The NPS identified national DUEs as a means to influence in-hospital drug use, and subsequently prescribing in the community. The NPS also sought to introduce some of the educational skills currently used in the community such as educational visiting (academic detailing) techniques and skills to a broader audience.

Despite the availability of Australian guidelines [4], knowledge of and adherence to the Community Acquired Pneumonia (CAP) management guidelines from the Australian Therapeutic Guidelines – Antibiotics (TGAB) in Australian hospitals is not optimal[5,6] Australia is fortunate to have the Therapeutic Guidelines, an independent organisation dedicated to developing guidelines for therapy from the latest world literature, interpreted and distilled by Australia's most eminent and respected experts [4]. The Therapeutic Guidelines – Antibiotics were first published in 1978, and are now available in the 13^th ^edition, in both hard copy and electronically [7]. The difficulty arises, as with many guidelines, in getting the best evidence actually translated into clinical practice.

The TGAB recommend treatment of CAP according to severity, as assessed by the Pneumonia Severity Index (PSI), a scoring system that was developed and validated by a large number of North American studies [8,9]. The TGAB includes a management algorithm, using the PSI severity class, to determine the most appropriate antibiotic choice and the most suitable site for management i.e. at home, at a general ward level or an intensive care unit [7,8].

The TGAB-recommended antibiotics were based on current microbiological trends in Australia. Streptococcus pneumonia is the most common cause of CAP in Australia [7] and although about 20% of clinical isolates have reduced susceptibility to penicillin, it is rare in Australia that the penicillin minimum inhibitory concentration exceeds 4 mg/L [10]. This differs to a number of other countries where resistance to penicillin is higher [11].

This manuscript provides details about the CAPTION experience in one state, QLD, specifically evaluating the implementation of the CAPTION project in QLD hospitals, detailing any benefits and drawbacks of involvement in a national drug use evaluation program.

## Methods

In 2004 Queensland (QLD) hospitals, public or private, with an Emergency Department, were deemed eligible and were invited to participate. Nine hospitals in QLD agreed. Approval from the Institutional Human Research Ethics Committees was sought where necessary, with some Committees requesting full ethical review.

The project employed established drug use evaluation (DUE) methodology, [12,13] previously successfully implemented across a number of other hospital projects by the QLD DUE group, NSW Therapeutic Advisory Group and the Victorian DUE Group previously. The CAPTION project involved data collection, and evaluation using the TGAB (12^th ^Ed) as the benchmark, feedback of evaluated data and targeted educational interventions. A baseline audit followed by two complete DUE cycles were implemented during the course of the 2-year project [12,13].

Each cycle involved the collection of data on 20 consecutive patients who presented to the ED with a provisional diagnosis of community-acquired pneumonia and were prescribed antibiotics while in the ED. Inclusion criteria were that the patient must be at least 18 years of age, and have a diagnosis of CAP documented in the medical record by ED doctors. It was assumed that the choice of empirical antibiotics was based on this diagnosis, and it was not judged whether the initial diagnosis was in fact correct.

Patients were excluded if they were less than 18 years of age; immunosuppressed; had cystic fibrosis; bronchiectasis; tuberculosis; aspiration or hospital acquired pneumonia; or had been discharged from hospital in the previous 14 days or transferred from another hospital.

This baseline data collection was followed by the implementation of a suite of interventions including academic detailing; provision of wall posters and PSI cards (to be attached to the identification (ID) badge) and group feedback presentations on results of the audit. This was followed by another period of data collection (audit two) and a second intervention and a final (third) audit.

Academic detailing training was provided to at least one person in each hospital. This person was nominated by the hospital, to provide the one-on-one education in that hospital. Training consisted of attendance at a two-day workshop, prior to the implementation of education, where the principles of social marketing were explained, and the opportunity provided to partake in role plays using these skills, with medical personnel from local emergency departments.

As each hospital completed data collection, the data (de-identified for patients) were sent to the QLD Project Coordinator and analysis conducted (de-identified for hospital). The proportion of patients for whom a PSI calculation had been recorded and the proportion of patients whose treatment was in concordance with the TGAB were determined using the Auditmaker^® ^program, programmed with the TGAB CAP management algorithm.

The implementation of the project was evaluated using feedback forms (Additional files 1 and 2) after each phase of the project (audit or intervention). Information collected included: duration of data collection period, number of records reviewed, ease of collection, time taken, and number of people educated, educational tools used and problems encountered during the education. Data was summarised by the state coordinator looking for themes. At the completion of the project a group meeting was held with the hospital coordinators during which positive and negative elements of the project were identified by individual hospitals using a presentation template, and general discussion of other benefits or drawbacks held.

The CAPTION project as a whole aimed to:

• Evaluate current practice with regards to management of CAP in Emergency Departments

• Introduce and implement the CAP management guidelines (TGAB 12^th ^Ed) into the emergency department

• Influence and improve the prescribing practice in the management of CAP in the emergency department

• Train hospital health care professionals in appropriate techniques for influencing and improving prescribing practice

## Results

### Data collection evaluation

Evaluation logs of data collection were completed by all hospitals after the first two audit cycles. In both audit cycles data were predominantly collected by pharmacists (11 occasions) but also included pharmacy students (6), medical staff (3) and students (1), registered nurse (3) and a pre-registration pharmacist (1). In both cycles some hospitals had multiple people involved in data collection, which may have resulted in some inconsistencies. Each hospital was provided with a manual which outlined specific details with regards to data collection, and regular contact was made by the state coordinator via email and teleconferences to answer any questions. All submitted data were checked by the state coordinator for anomalies and corrected before analysis. Problems encountered were similar across all sites and both audits and included:

• Access to records

• Legibility of Dr's writing

• Inadequate information recorded in the chart

Time required collecting the data varied, with the average time to review a record & collect data was initially 28 minutes. This decreased in the second audit to 15 minutes, as data collectors became familiar with the medical record and the requirement of data collection.

### Intervention Evaluation

Sixteen of eighteen possible evaluation logs were returned after the completion of the two intervention cycles. These logs indicated that implementation of the academic detailing intervention was difficult at a number of sites, with at least two sites being unable to implement academic detailing at various times due to time constraints and staffing levels. Where detailing occurred it was however found that most doctors were really appreciative of someone taking the time to talk to them. This was especially relevant in the ED where visits by pharmaceutical representatives may be less frequent, and not involve junior staff. The detailers found that the detailing cards (available upon request from authors) were very useful, as a guide to lead them through the interaction as well as keeping the message consistent; however the detailers did find that not all staff wanted to keep them.

The barrier to the implementation of academic detailing in the Emergency Department most commonly identified was time. Time was an issue for the person conducting the educational visits. It was difficult to make an appointment with Emergency Department doctors. Another concern was the lack of available appropriate space to conduct a one-on-one educational visit in a busy Emergency Department. Additionally some Senior Emergency physicians also were not supportive of one-on-one education.

Across the two intervention periods, a total of 185 staff members in QLD were visited by CAPTION trained local hospital project officers, with an average duration of 12.6 minutes. A mean of 12 staff members per hospital were detailed, with the majority being to resident medical officers, followed by registrars and physicians. Sixteen group education sessions were conducted, reaching a total of 208 staff members.

Comments received regarding the intervention included:

"*Many doctors, particularly the junior doctors, appeared to be appreciative of the session, and the information provided"*

"Academic detailing; Thankful for input of pharmacist's time"

"Due to the busy nature of the ED, it is very difficult to capture significant number of doctors to attend a group presentation"

*"Found that detailing is very time consuming and would be easier to give a group session"*.

### Evaluation of Project overall

In the final QLD CAPTION group wrap-up meeting, participants from all nine hospitals undertook individual hospital presentations, outlining their perceptions of the benefits of the project. These benefits are grouped into the impact on the hospital dynamic such as improved interdisciplinary working e.g. between pharmacist and doctor; new roles for pharmacist in the ED; recognition of educational/academic role of the pharmacist and involvement with the NPS and personal benefits. Participants described personal benefits such as receiving academic detailing training, improved communication skills to use when aiming to influence prescribing, and the opportunity to be involved in project work and resultant presentations personally rewarding. The drawback of participation is the extra burden on already busy staff members, and time commitments which may have made it difficult to give the CAPTION project the attention warranted.

Positive and negative elements of participation were reflected by participants:

"Essential Skills learned in influencing prescribing habits through Academic Detailing."

"Really feels like we are making a difference in assisting doctor's prescribing when seeing doctors calculating PSI and prescribing based on Therapeutic Guideline recommendations."

"It was found that most doctors were really appreciative of someone taking the time to talk to them"

"Creating a pharmacy presence in the Emergency department"

"Resource Materials and involvement with the NPS on a nationwide basis"

"It was a good learning curve and I enjoyed the experience!!!"

"Time consuming"

"Try to track down doctors who were either busy or working on late shifts, and get them to sit down for 10 minutes to have an academic detailing session!!!"

"Co-ordinating time, staff, health records, doing own ward work AND being short of pharmacy staff"

The participants acknowledged the amount of extra work that was required to participate in CAPTION, however were appreciative of the personal rewards in participation in a national multicentre project.

Where there was support at a District Manager level in the CAPTION hospitals the participants found that the project had wider acceptance. Other components reported by CAPTION participants as impacting on the project were the improved relationships between pharmacy and medical staff and the training undertaken in academic detailing. These were considered a major benefit of the project.

## Discussion

### Intervention Evaluation

A number of methods have been described in the literature with respect to changing prescriber behaviour, with varying levels of evidence about effectiveness. Interventions that have shown some evidence for change include audit and feedback [14] where response is usually better when baseline concordance to recommendations is initially low. Group education sessions delivered in an interactive format have also been shown to change behavior, but didactic education sessions are unlikely to have any effect. [15] The CAPTION project used a suite of interventions including audit, feedback and group presentations, with the principle method of behaviour change being academic detailing. This technique, also known as educational visiting, which has been extensively reviewed [16-18] and is commonly used in community practice in Australia [3]. Until the CAPTION project however, the technique had not been formally implemented within Australia's hospital system. Reasons for this are the intensive training required (minimum two day workshop), high turnover of staff and frequent rotation of medical staff. It has been perceived in the past that this therefore requires repetition of training and repetition of the actual educational visit with new staff, which has not been considered to be an efficient or effective use of resources. Academic detailing to date has been evaluated only in community practice, therefore data are still lacking on the effectiveness of this educational technique in a hospital setting.

In community practice in Australia, divisions of General Practice employ facilitators to undertake educational visiting (academic detailing) on behalf of the NPS, providing visits of up to 40 minutes in duration. This duration would be impractical in the hospital environment and presented another barrier, and thus educational visits for this CAPTION program were specifically tailored to last approximately 10 minutes.

It was identified that there was some difficulty in making appointments with Emergency Department doctors due to the unpredictable nature of their workload. Group education sessions in this setting were thus considered a more suitable option. However academic detailing, when performed in conjunction with other interventions proved to be a useful educational strategy to change behaviour within the Emergency Department in CAPTION.

A study from Germany [19] that evaluated the quality circle programmes to implement clinical guidelines in general practice found that for doctors who participated in the programme there was an increase in knowledge, improved work relationships and was beneficial beyond actual measured clinical care for patients. In the CAPTION project the hospital coordinators reported that the benefit of participation went beyond improving patient care and also improved their inter-professional relationships and their knowledge and was beneficial both personally and to the hospital. An additional paper looking at decreasing in-hospital mortality, reported on components that had the most influence [20]. This demonstrated that leadership by the hospital executive contributed to the success of the strategy for change. Similarly where there was support at a District Manager level in the CAPTION hospitals the participants found that the project had wider acceptance.

Hospitals may not undertake such projects individually due to the time commitments, including the preparation of audit materials and preparation of education materials, unless there is a designated project staff member such as a DUE pharmacist. A national multicentre drug use evaluation project such as CAPTION allows hospitals that would otherwise not undertake such a project the opportunity to participate. All materials were developed at a national level resulting in a sharing of resources. Participation in a multicentre project allows hospitals to benchmark their own institution's data against state and national results. In Australia we are fortunate to have the Therapeutic Guidelines, which assist in obtaining consensus about treatment guidelines and facilitates multicentre projects. The QLD arm of CAPTION resulted in other benefits to the individual participants and their hospitals. These benefits demonstrated the additional value of participating in a multicentre project of this type.

This paper reports the Queensland experience of participation in a national quality improvement initiative. This program was conducted concurrently in New South Wales, Victoria, South Australia and Tasmania, coordinated nationally by the NPS. Similar experiences were reported by the other states. The challenge in implementing this type of program nationally is firstly obtaining consensus. Not all physicians believe in the PSI, due to its perceived complexity using 19 different variables, the incorporation of arterial blood gases in the measurement and the weighting due to age. Some hospitals believe that the CURB [21] or CURB-65 [22] scores are easier to use. The Therapeutic Guidelines: Antibiotic is well accepted in Australia as a consensus document. Implementation in other institutions or health systems may be more difficult without a national coordinating body such as the NPS or without firstly obtaining consensus for managing the patient and choice of antibiotic.

It is however possible to apply the process discussed in this paper to other health systems, using either the PSI or another scoring system and conduct audit and feedback along with an educational intervention incorporating one-on-one education. Appropriate training in the educational techniques would be required. The additional challenges of implementing such a program such as time, workload and training remain the same in any health system.

## Conclusion

Projects which aim to improve specific disease outcomes, such as implementation of guidelines for the optimal management of community acquired pneumonia, are often perceived as difficult, of minimal benefit to the institution and not successful in achieving long lasting changes. Participation in a multicentre DUE demonstrated many benefits to the institutions and individuals in addition to improvements in prescribing.

## Competing interests

The authors declare that they have no competing interests.

## Authors' contributions

SET, JC conceived the project. LKP, SET and JC participated in its design and coordination. LKP collected the data. LKP and SET drafted the manuscript. All authors read and approved the final manuscript.

## Pre-publication history

The pre-publication history for this paper can be accessed here:



## Supplementary Material

Additional file 1 **Data collection log audit1**. A sample of the evaluation log used after each audit cycle. Click here for file

Additional file 2 **Intervention activity log**. A sample of the evaluation log used after each intervention cycle. Click here for file

## References

[B1] Pulver LK, Tett SE (2006). Drug utilization review across jurisdictions – a reality or still a distant dream?. Eur J Clin Pharmacol.

[B2] Maxwell DJ, McIntosh KA, Pulver LK, Easton KL (2005). Empiric management of community-acquired pneumonia in Australian emergency departments. Medical Journal of Australia.

[B3] Weekes LM, Mackson JM, Fitzgerald M, Phillips SR (2005). National Prescribing Service: creating an implementation arm for national medicines policy. Br J Clin Pharmacol.

[B4] Therapeutic Guidelines. http://www.tg.com.au/.

[B5] Robertson MB, Korman TM, Dartnell JGA, Ioannides-Demos LL, Kirsa SW, Lord JAV, Munafo L, Byrnes GB (2002). Ceftriaxone and cefotaxime use in Victorian hospitals. Medical Journal of Australia.

[B6] Robertson MB, Korman TN, Dartnell JGA, Ioannides-Demos LL, Kirsa SW, Lord JAV (2002). Treatment of lower respiratory tract infection in emergency departments. Australian Society for Antimicrobials 3rd Annual Scientific Meeting 2002; Sydney.

[B7] Antibiotic Writing Group (2003). Therapeutic guidelines: antibiotic.

[B8] Fine MJ, Smith MA, Carson CA, Mutha SS, Sankey SS, Weissfeld LA, Kapoor WN (1996). Prognosis and outcomes of patients with community-acquired pneumonia. A meta-analysis. JAMA: the journal of the American Medical Association.

[B9] Fine MJ, Auble TE, Yealy DM, Hanusa BH, Weissfeld LA, Singer DE, Coley CM, Marrie TJ, Kapoor WN (1997). A prediction rule to identify low-risk patients with community-acquired pneumonia. New England Journal of Medicine.

[B10] Johnson PD, Irving LB, Turnidge JD (2002). Community-acquired pneumonia. Medical Journal of Australia.

[B11] Collignon PJ, Turnidge JD (2000). Antibiotic resistance in Streptococcus pneumonia. Medical Journal of Australia.

[B12] Doherty P, Kirsa SW, Chao S, Wiltshire S, McKnight D, Maxwell DJ, Dartnell JG, Kaye K, Graudins L (2004). SHPA Standards of Practice for Drug Usage Evaluation in Australian Hospitals. Journal of Pharmacy Practice and Research.

[B13] COSP in DUE (2004). Australian Drug Usage Evaluation (DUE) Starter Kit.

[B14] Jamtvedt G, Young JM, Kristoffersen DT, O'Brien MA, Oxman AD (2006). Audit and feedback: effects on professional practice and health care outcomes. Cochrane Database of Systematic Reviews.

[B15] Thomson O'Brien MA, Freemantle N, Oxman AD, Wolf F, Davis DA, Herrin J (2001). Continuing education meetings and workshops: effects on professional practice and health care outcomes. Cochrane Database of Systematic Reviews.

[B16] Avorn J, Soumerai SB (1983). Improving drug-therapy decisions through educational outreach. A randomized controlled trial of academically based "detailing". New England Journal of Medicine.

[B17] Soumerai SB, Avorn J (1990). Principles of educational outreach ('academic detailing') to improve clinical decision making. JAMA: the journal of the American Medical Association.

[B18] O'Brien MA, Rogers S, Jamtvedt G, Oxman AD, Odgaard-Jensen J, Kristoffersen DT, Forsetlund L, Bainbridge D, Freemantle N, Davis DA (2007). Educational outreach visits: effects on professional practice and health care outcomes. Cochrane Database of Systematic Reviews.

[B19] Tausch BD, Harter MC (2001). Perceived effectiveness of diagnostic and therapeutic guidelines in primary care quality circles. Int J Qual Health Care.

[B20] Wright J, Dugdale B, Hammond I, Jarman B, Neary M, Newton D, Patterson C, Russon L, Stanley P, Stephens R (2006). Learning from death: a hospital mortality reduction programme. J R Soc Med.

[B21] British Thoracic Society Standards of Care Committee (2001). BTS Guidelines for the Management of Community Acquired Pneumonia in Adults. Thorax.

[B22] Lim WS, Eerden MM van der, Laing R, Boersma WG, Karalus N, Town GI, Lewis SA, Macfarlane JT (2003). Defining community acquired pneumonia severity on presentation to hospital: an international derivation and validation study. Thorax.

