# Supplementary material for: The Queensland experience of participation in a national drug use evaluation project, Community-acquired pneumonia – towards improving outcomes nationally (CAPTION)
Source: BMC Pulm Med. 2009 Aug 3;9:38. doi: 10.1186/1471-2466-9-38 (PMC2731033; doi:10.1186/1471-2466-9-38)
Supplement: Additional file 1 — Data collection log audit1. A sample of the evaluation log used after each audit cycle. [file 1471-2466-9-38-S1.pdf]

CAPTION PROJECT

Community-Acquired Pneumonia: Towards Improving Outcomes Nationally

DATA COLLECTION LOG (1st AUDIT cycle)

This log summarises the CAPTION data collection process and any issues encountered. Please complete after the review of patient records and return to your State Project Officer. Please remember, your responses will be kept strictly confidential. We appreciate your open and honest feedback

Hospital ID _ _ _

Data collection start date: — — /— — / — —

Data collection end date: — — /— — / — —

Name of person doing data collection (optional) __________________

Profession of person(s) doing data collection _____________ _____________ _____________

Total number of records reviewed _ _ _ Number of records discarded _ _ _

How was data collection carried out? (please circle)

From paper then entry into Audit Maker *Directly to Audit Maker* *Combination of both*

Average time taken per record (mins) _ _ _

Please record any particular problems in reviewing records (e.g. access to records/legibility/inadequate information recorded etc.)

____

____

Please rate (by circling) on the following 5-point scale how easy Audit Maker was to use, where 1 equals extremely easy and 5 equals extremely difficult:

1	2	3	4	5
Extremely easy				*Extremely difficult*

Please record any particular problems or comments related to either the data collection process and/or using Audit Maker (in particular any problems with data entry and/or generating the feed-back report):

____

____

Thank you for your feedback.

Please return your completed log to your State project officer.
