# Supplementary material for: The Queensland experience of participation in a national drug use evaluation project, Community-acquired pneumonia – towards improving outcomes nationally (CAPTION)
Source: BMC Pulm Med. 2009 Aug 3;9:38. doi: 10.1186/1471-2466-9-38 (PMC2731033; doi:10.1186/1471-2466-9-38)
Supplement: Additional file 2 — Intervention activity log. A sample of the evaluation log used after each intervention cycle. [file 1471-2466-9-38-S2.pdf]

CAPTION PROJECT

Community-Acquired Pneumonia: Towards Improving Outcomes Nationally

Intervention activity log

This log provides the CAPTION project with a description of how the CAP educational intervention was carried out in your hospital. Please complete and return to your State Project Officer.

To be completed by hospital coordinator:

Hospital ID ___________________________

Please complete for as many group session(s) as were carried out:

ED Staff	Group session 1 - Number of staff participating	Group session 2 - Number of staff participating	Group session 3 - Number of staff participating	How many staff attended at least one session?
**Date**	_ _/_ _/_ _	_ _/_ _/_ _	_ _/_ _/_ _	
**Doctors**				
Interns				
Resident Medical Officer				
Registrar				
Physicians/Staff specialist				
General Practitioners				
Other Medical Practitioner				
**Nurses**				
Nurse unit manager				
Clinical nurse consultant/educator				
Clinical nurse specialist				
Registered nurse				
Enrolled nurse				
**ED (other)**				
ED pharmacist				
Other (specify)				

Average duration of group sessions? _________

Please complete for one-on-one sessions:

ED Staff	Number of staff who had a one-on-one session	Average duration of session
**Doctors**		
Interns		
Resident Medical Officer		
Registrar		
Physicians/Staff specialist		
General Practitioners		
Other Medical Practitioner		
**Nurses**		
Nurse unit manager		
Clinical nurse consultant/educator		
Clinical nurse specialist		
Registered nurse		
Enrolled nurse		
**ED (other)**		
ED pharmacist		
Other (specify)		

Did any staff receive >1 one-on-one education session? *Yes* *No*

Please explain which staff received >1 one-on-one education session(s) and why?

____

____

Was the detailing card used in one-on-one education sessions? (please circle): *Always* *Sometimes* *Never*

Was power-point template used in group education sessions (please circle): *Yes* *No*

Number of posters distributed in ED ________ Number of leaflets distributed in ED ________

Number of other educational materials distributed in ED -please specify type/number : ____________________

Any problems/barriers/solutions encountered during the educational sessions?

____

____

Thank you for your feedback.

Please return your completed log to your State project officer
